# The performance of VCS(volume, conductivity, light scatter) parameters in distinguishing latent tuberculosis and active tuberculosis by using machine learning algorithm

**DOI:** 10.1186/s12879-023-08531-2

**Published:** 2023-12-16

**Authors:** Lijiao Chen, Lingke Yuan, Tingting Sun, Ruiqing Liu, Qing Huang, Shaoli Deng

**Affiliations:** 1 Department of Laboratory Medicine, Daping Hospital, Army Medical University, Chongqing, 400042 P.R. China; 2 Science in Computational Finance, Carnegie Mellon University, Pittsburgh, PA USA; 3 College of Medical Technology, Chongqing Medical and Pharmaceutical College, Chongqing, China

**Keywords:** Leukocyte VCS (volume, conductivity, light scatter) parameters, Machine learning algorithm, Active tuberculosis infection, Latent tuberculosis infection, Distinguish

## Abstract

**Background:**

Tuberculosis is a chronic infectious disease caused by mycobacterium tuberculosis (MTB) and is the ninth leading cause of death worldwide. It is still difficult to distinguish active TB from latent TB,but it is very important for individualized management and treatment to distinguish whether patients are active or latent tuberculosis infection.

**Methods:**

A total of 220 subjects, including active TB patients (ATB, *n* = 97) and latent TB patients (LTB, *n* = 113), were recruited in this study .46 features about blood routine indicators and the VCS parameters (volume, conductivity, light scatter) of neutrophils(NE), monocytes(MO), and lymphocytes(LY) were collected and was constructed classification model by four machine learning algorithms(logistic regression(LR), random forest(RF), support vector machine(SVM) and k-nearest neighbor(KNN)). And the area under the precision-recall curve (AUPRC) and the area under the receiver operating characteristic curve (AUROC) to estimate of the model’s predictive performance for dentifying active and latent tuberculosis infection.

**Results:**

After verification,among the four classifications, LR and RF had the best performance (AUROC = 1, AUPRC = 1), followed by SVM (AUROC = 0.967, AUPRC = 0.971), KNN (AUROC = 0.943, AUPRC = 0.959) in the training set. And LR had the best performance (AUROC = 0.977, AUPRC = 0.957), followed by SVM (AUROC = 0.962, AUPRC = 0.949), RF (AUROC = 0.903, AUPRC = 0.922),KNN(AUROC = 0.883, AUPRC = 0.901) in the testing set.

**Conclusions:**

The machine learning algorithm classifier based on leukocyte VCS parameters is of great value in identifying active and latent tuberculosis infection.

## Introduction

Tuberculosis is a chronic infectious disease caused by mycobacterium tuberculosis (MTB) and is the ninth leading cause of death worldwide. Since 2014 Tuberculosis disease (TB) has surpassed Acquired Immunodeficiency Syndrome (AIDS) as the leading cause of death from a single infectious agent [1, 2]. TB annually worldwide.China is one of the top 22 countries with high tuberculosis (TB) burden, ranking 3nd in the world. World Health Organization (WHO) global TB report 2020 (WHO, 2020) stated that the TB mortality could increase to the levels seen in 2015 or even 2012 impacting of the COVID-19 pandemic on global. Therefore, more attention should be paid to the prevention and control of the disease under the current global severe COVID-19 pandemic situation [3]. Prompt diagnosis and early initiation of treatment remain key strategies in TB prevention and control.

At present, the existing tuberculosis laboratory diagnosis cannot meet the clinical needs. Acid-fast staining and bacterial culture are the gold standards for tuberculosis diagnosis, but they have the disadvantages of low positive rate and time-consuming. Although PCR (polymerase chain reaction) analysis has high sensitivity and specificity, it cannot be widely used for diagnosis in primary medical institution due to expensive laboratory hardware facilities and restrictions on the types of clinical specimens. Interferon-gamma release assay (IGRAs) has high sensitivity and specificity for tuberculosis infection, but it has a shortcoming that cannot distinguish between latent tuberculosis infection and active tuberculosis [4]. Our team found that the monocyte-related indicators in the VCS parameters of tuberculosis patients changed significantly in the previous study. The three indicators of mean monocyte volume (MMV), mean monocyte volume standard deviation (MMV-SD), and mean monocyte conductivity (MMC) can be combined to obtain superior diagnostic performance(sensitivity: 93.8%, specificity: 93.1%) [5]. These indicators can be used as auxiliary indicators to differentiate between active pulmonary tuberculosis and latent tuberculosis infection.

In recent years, many scholars have explored new models of disease diagnosis based on big data and machine learning algorithms in the medical field,and have achieved remarkable results in disease risk prediction and diagnosis [6–8]. Artificial intelligence (AI) using machine learning (ML) is an ensemble of techniques that automatically learn patterns from data and that require no assumptions regarding the structure of the data. A strength of these techniques is that they capture non-linear relationships in the data, as well as interaction between predictors. Many studies have demonstrated their promising performance for diseases prediction [9].

In this study, we aimed to evaluate the performance of VCS parameters in distinguishing latent tuberculosis and active tuberculosis by using machine learning algorithms.

## Materials and methods

### Data collection

We retrospectively analyzed the VCS parameters of neutrophils, monocytes, and lymphocytes from 97 active Tuberculosis patients (Group:ATB) and 113 latent Tuberculosis patients (Group:LTB) by using a hematology analyzer with VCS (volume, conductivity, light scatter) technology from January 2018 to July 2018 in Chongqing Infectious Disease Medical Center and Army Medical Center (Daping Hospital), Army Medical University, Chongqing, China. All patients were diagnosed for the first time who had never had treatment for TB before or has not yet initiated anti-TB treatment.

The inclusion criteria for each group were as follows: ATB was diagnosed based on typical clinical symptoms, and/or chest X-ray findings in line with tuberculosis imaging lesions, and/or a molecular test (Xpert MTB/RIF, Cepheid, Sunnyvale, CA, USA), for the most important positivity on at least one of the following tests: acid-fast bacilli or bacterial culture. These individuals had no previous history of TB disease or treatment. LTB cases were defined as those who has a history of TB exposure and with a positive interferon-gamma release assay T-SPOT (Oxford Immunotec, Oxfordshire, UK) test, sputum smear and MTB culture were negative, and the absence of clinical and radiographic signs of ATB. All samples were collected in EDTA-anti-coagulated tubes and analyzed within 6 h after specimen were collected by a hematology analyzer with VCS technology. The VCS parameters of neutrophils(NE), monocytes(MO), and lymphocytes(LY) included mean volume (MV) and its deviation(MV-SD), mean conductivity (MC) and its deviation(MC-SD), multiple light scatters like median angle light scatter(MALS), upper median angle light scatter(UMALS), lower median angle light scatter(LMALS), low-angle light scatter(LALS), axial light loss(AL2) and their deviation(MALS-SD, UMALS-SD, LMALS-SD, LALS-SD, AL2-SD) were collectioned and analysised,also including some routine indicators (e.g. total leukocyte count(WBC) and the percentage of neutrophil, monocyte, and lymphocyte(NE%,MO%,LY%)). Thus a total of 46 parameters were obtained.

### Analysis of baseline features and evaluate performance of peripheral blood routine parameters and the VCS parameters of neutrophils, monocytes, and lymphocytes in distinguishing latent tuberculosis and active tuberculosis

Continuous variables were described as mean ± SD or median (Q1 and Q3). Comparison between two groups was performed by the Student’s t test or Wilcoxon rank-sum test according to whether the data conformed to a normal distribution. Comparison of the gender differences between groups was performed using the Chi-squared test. Categorical variables were expressed as as composition ratio or rate%), and comparison was made by Chi-square test. Analyses were conducted using Software version 27.0 (Social Sciences Inc, Chicago, Illinois, USA).

### Classifier

In this study, four machine-learning classification algorithms were used namely, LR (logistic regression),RF(random forest), KNN(K-nearest neighbor) and SVM(supportive vector machine) by using Python3 and running on Windows. A brief description of each method is provided in the following paragraphs.Data obtained from participants in discovery cohort were randomly divided at 8:2 ratio. The larger (8/10) one was applied for modeling (training set), whereas the smaller one (2/10) was used as test set.

### LR (logistic regression)

Logistic regression is a common Machine Learning algorithm for binary classification. It is a linear model for classification, which can fit binary or multinomial logistic regression.

### RF(random forest)

Random Forest (RF) is based on decision trees, proposed by Breiman, is a supervised, non-parametric method of classification. It is an ensemble classifier used for data mining, and is composed of numerous decision trees, each one relying on the values of a random vector sampled independently. By using random subsets of the training data for each tree and considering random features for each decision point, Random Forest prevents over fitting.

### KNN(K-nearest neighbor)

K-nearest neighbor (Cover and Hart, 1967) is a simple classification algorithm, and it is also called Reference Sample Plot Method. The idea of this *algorithm* is when given an unknown sample, a k-nearest neighbor (KNN) classifier searches a feature space for the k training samples that are closest to the unknown sample. This means KNN algorithm predicts the class of a sample with unknown class by considering the classes of k-nearest neighbors.

### SVM(supportive vector machine)

SVM is developed by Corinna Cortes and Vapnik, the core of which is the structural risk minimum principle constructed by the empirical risk minimum principle and confidence intervals.

### Model building, model performance evaluation and validation

**Fig. 1:**
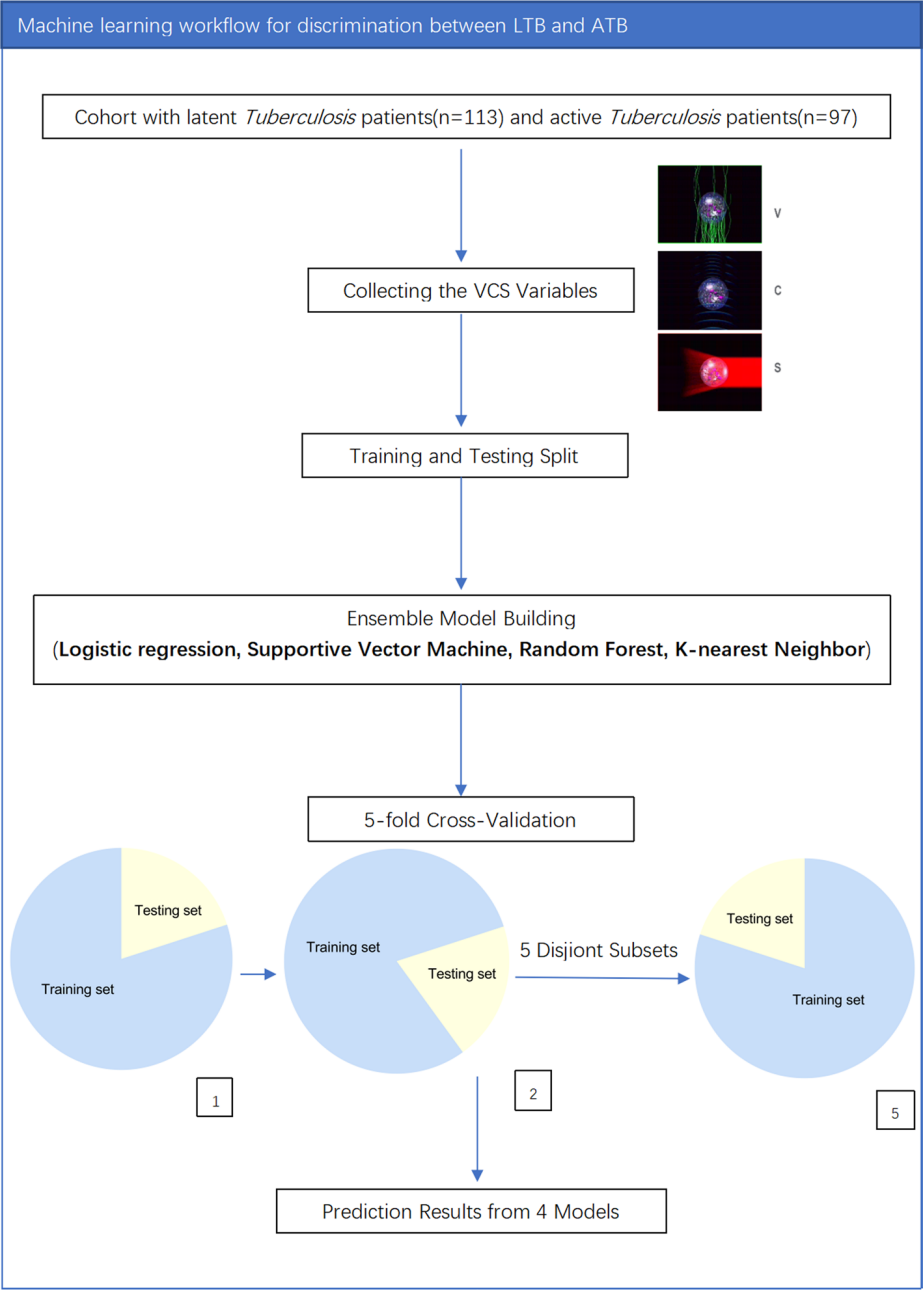
Machine learning workflow for discrimination between LTB and ATB

In the training set, k-fold cross-validation (k = 5) was used, all 210 subjects were randomly divided into 5 equally sized subsets. Firstly, we used four of them in turn as the training set and one as the test set. k-Fold cross validation (k = 5)repeats this steps 5 times changing a partition serving as a test set one by one. In the end, averaged predictive performance over k validation steps is regarded as the predictive performance of a classification algorithm (Fig. 1). We evaluated diagnostic ability of each model based on follow indexes: accuracy, precision, recall, F1 SCORE, Matthews correlation coefcient(MCC), Specifcity and negative predictive value(NPV). The calculation formulas of these indexes were shown in a table (Table 1). We plotted the area under the precision-recall curve (AUPRC) and the area under the receiver operating characteristic curve (AUROC) to compare the performances of the machine learning classification models. In the testing set, we also plotted the area under the precision-recall curve (AUPRC) and the area under the receiver operating characteristic curve (AUROC) to estimate of the model’s predictive performance.

**Table 1:** The calculation formula for the evaluation index in the training set

evaluation index	calculation formula
Accuracy	$\frac{TP+TN}{TP+FP+TN+FN}$
Precision	$\frac{TP}{TP+FP}$
Recall	$\frac{TP}{TP+FN}$
F1 SCORE	$\frac{2\ast precesion\ast recall}{precesion+recall}$
MCC	$\frac{TP\ast TN-FP\ast FN}{\sqrt{\left(TP+FP\right)\left(TP+FN\right)\left(TN+FP\right)\left(TN+FN\right)}}$
Secifcity	$\frac{TN}{TN+FP}$
NPV	$\frac{TN}{TN+FN}$

*Abbreviations*: *MCC* Matthews correlation coefcient, *NPV* negative predictive value, *TP* true positive, *FP* false positive, *TN* true negative, *FN* false negative

## Results

### Demographic characteristic and peripheral blood parameters of the participants

**Fig. 2:**
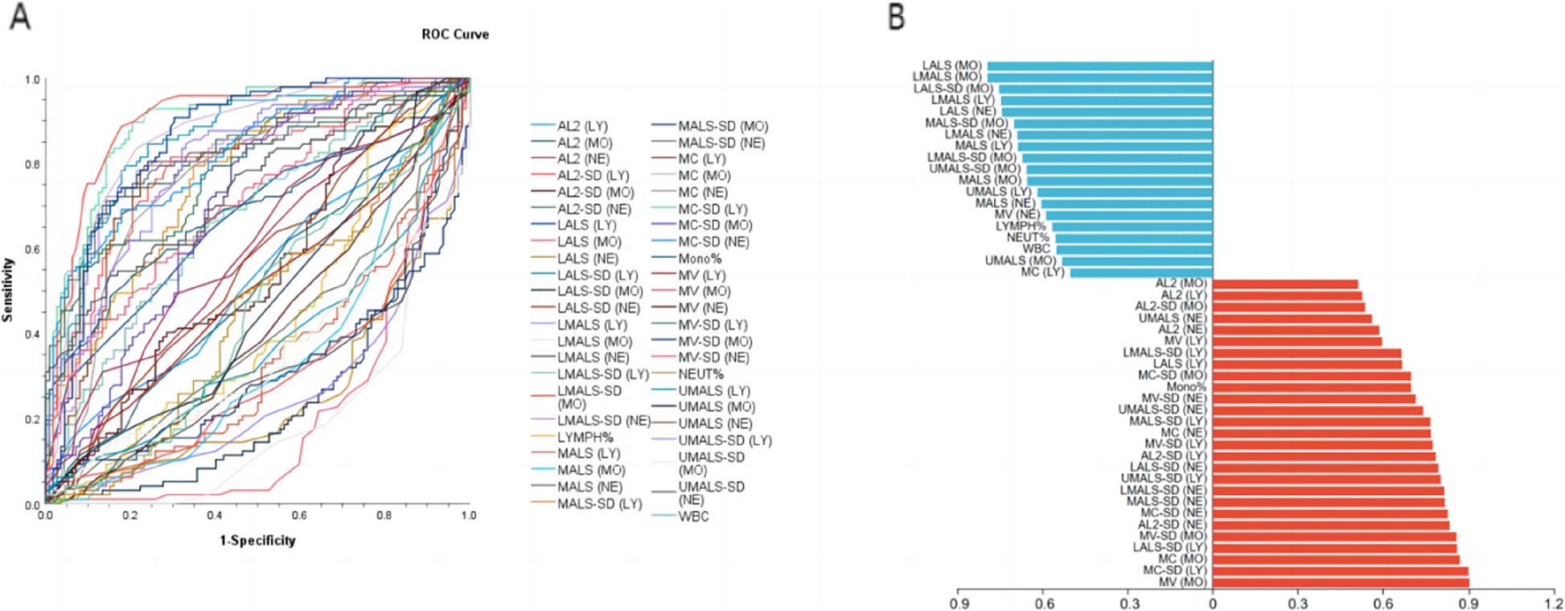
Performance of 46 features about blood routine indicators and the VCS parameters in differentiating ATB patients from LTBI individuals. **A** ROC curves showing the diagnostic performance of 46 features in discriminating between ATB and LTBI. Curves in the upper indicated that the levels of these indicators are higher in ATB group than in LTBI group. Curves in the bottom indicated that the levels of these indicators are lower in ATB group than in LTBI group. **B** Bar graphs showing the AUC of various indicators in discriminating ATB patients from LTBI individuals.The red parts represent area under the ROC curve(AUC) when group ATB as the state variable to plot the ROC curve and the red parts represent area under the ROC curve(AUC) when group LTBI as the state variable to plot the ROC curve. ATB, active tuberculosis; LTBI, latent tuberculosis infection; ROC, receiver operator characteristics; AUC, area under the ROC curve

The retrospectively study was conducted on a total of 97 active pulmonary tuberculosis patients (male/female: 60/37; mean age: 49.80 ± 14.50) and 113 latent tuberculosis infection patients (male/female: 46/67; mean age: 51.45 ± 17,57), with statistically significant difference in gender (*p* = 0.002). In addtion, there were statistically remarkable differences between the two groups because the AUC including of MV (MO),MC-SD (LY),MC (MO),LALS-SD (LY),MV-SD (MO),AL2-SD (NE),MC-SD (NE),MALS-SD (NE),LMALS-SD (NE) and UMALS-SD (LY) were greater than 0.8 in discriminating between ATB and LTBI (Tables 2 and 3, Fig. 2).

**Table 2:** The area under the ROC curve (AUC) and 95% confidence interval of the AUC for 46 parameters for distinguishing latent tuberculosis and active tuberculosis

Parameter	area under the ROC curve (AUC)	95% confidence interval
MV (MO)	0.903	0.858–0.947
MC-SD (LY)	0.9	0.858–0.942
MC (MO)	0.87	0.821–0.919
LALS-SD (LY)	0.859	0.808–0.909
MV-SD (MO)	0.857	0.805–0.908
AL2-SD (NE)	0.833	0.778–0.889
MC-SD (NE)	0.827	0.772–0.881
MALS-SD (NE)	0.817	0.757–0.876
LMALS-SD (NE)	0.815	0.755–0.874
UMALS-SD (LY)	0.802	0.742–0.861
LALS-SD (NE)	0.794	0.733–0.856
AL2-SD (LY)	0.784	0.719–0.848
MV-SD (LY)	0.774	0.710–0.839
MC (NE)	0.768	0.703–0.834
MALS-SD (LY)	0.766	0.702–0.829
UMALS-SD (NE)	0.741	0.674–0.808
MV-SD (NE)	0.714	0.645–0.783
Mono%	0.698	0.626–0.771
MC-SD (MO)	0.698	0.628–0.769
LALS (LY)	0.667	0.592–0.741
LMALS-SD (LY)	0.664	0.591–0.738
MV (LY)	0.596	0.518–0.673
AL2 (NE)	0.586	0.508–0.663
UMALS (NE)	0.56	0.482–0.637
AL2-SD (MO)	0.536	0.457–0.615
AL2 (LY)	0.526	0.446–0.605
AL2 (MO)	0.511	0.432–0.590
MC (LY)	0.502	0.422–0.581
UMALS (MO)	0.531	0.452–0.61
WBC	0.551	0.472–0.629
NEUT%	0.554	0.476–0.632
LYMPH%	0.565	0.486–0.643
MV (NE)	0.587	0.51–0.664
MALS (NE)	0.604	0.527–0.682
UMALS (LY)	0.617	0.54–0.695
MALS (MO)	0.654	0.579–0.728
UMALS-SD (MO)	0.656	0.581–0.732
LMALS-SD (MO)	0.67	0.597–0.743
MALS (LY)	0.686	0.613–0.759
LMALS (NE)	0.688	0.615–0.762
MALS-SD (MO)	0.7	0.627–0.773
LALS (NE)	0.742	0.673–0.81
LMALS (LY)	0.746	0.678–0.813
LALS-SD (MO)	0.753	0.687–0.819
LMALS (MO)	0.793	0.731–0.854
LALS (MO)	0.793	0.732–0.855

*Abbreviations*: *MV* mean volume, *MC* mean conductivity, *MALS* median angle light scatter, *UMALS* upper median angle light scatter, *LMALS* lower median angle light scatter, *LALS* low-angle light scatter, *AL2* axial light loss, *WBC* leukocyte count, *NE%* the percentage of neutrophil, *LY%* the percentage of lymphocyte, *MO%* the percentage of monocyte

**Table 3:** Baseline features of the participants

baseline feature	active pulmonary tuberculosis(ATB)	latent tuberculosis infection(LTB)	p
Gender (male/female)	60/37	46/67	*0.002*
Age (years)	49.80 ± 14.50	57.14 ± 14.4	*0.002*
T.SPOT	97/97	113/113	N/A
Acid-fast staining	51/97	0/113	N/A
Positive culture for MTB	46/97	0/113	N/A

*Abbreviations*: *MTB* mycobacterium tuberculosis, *N/A* not applicable

### Performance of different models

**Fig. 3:**
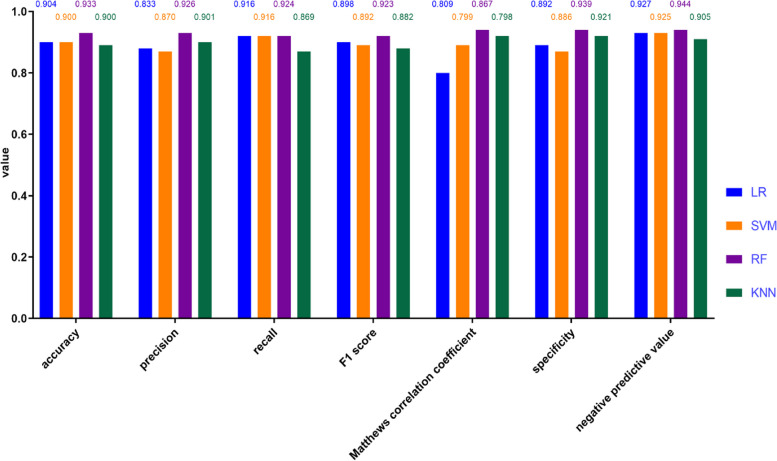
The efficacy of LR,SVM,RF and KNN in the training set

In this study, the developed machine learning models for classificationand regression are evaluated with the following metrics:accuracy, precision, recall, F1 SCORE, Matthews correlation coefcient(MCC), Specifcity and negative predictive value(NPV), the area under the precision-recall curve( AUPRC), the area under the receiver operating characteristic curve (AUROC). To improve the model performance, some parameters were optimized (Table 4). The accuracy values of LR, SVM, RF and KNN were 0.904, 0.900, 0.933, 0.900. All models had accuracy values equal to or greater than 0.900. The precision values of LR, SVM, RF and KNN were 0.883,0.870,0.926,0.901. And the RF model obtained the highest precision (0.926) and following KNN with a second best precision(0.901). The results indicated that RF was the best-performing model in the training set because it obtained the highest accuracy(0.933), precision(0.926),recall(0.924),F1 SCORE(0.923), Matthews correlation coefcient(MCC)(0.867), Specifcity(0.939) and negative predictive value(NPV)(0.944) (Fig. 3).

**Table 4:** The parameters selection in the predictive models

Models	Tuning parameter
LR	Penalty = l2 C (Cost) = 100 max_iter = 100 intercept_scaling = 10
SVM	Probability = True Kernel = linear Max_iter = 100
RF	Random_state = 42 N_estimators = 150
KNN	N_neighbors = 3

*Abbreviations*: *LR* logistic regression, *SVM* support vector Machiner, *RF* random forest, *KNN* k-nearest neighbors classifier

**Fig. 4:**
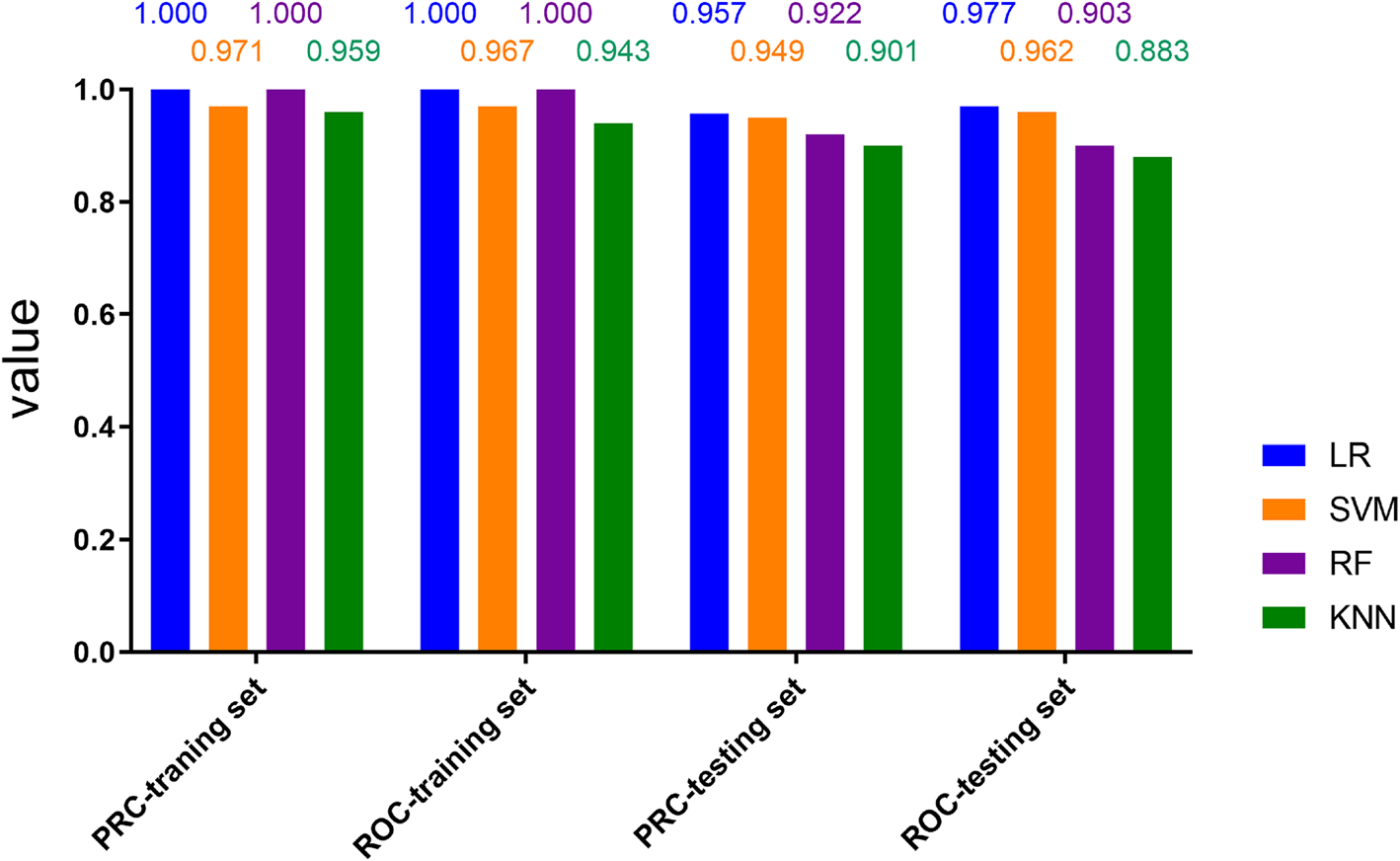
The area under the precision-recall curve ( AUPRC) and area under the receiver operating characteristic curve (AUROC) for discrimination between LTB and ATB in traning set and testing set

Among the four classifications, LR and RF had the best performance (AUROC = 1, AUPRC =1), followed by SVM (AUROC = 0.967, AUPRC =0.971), KNN (AUROC = 0.943, AUPRC =0.959) in the training set. And LR had the best performance (AUROC = 0.977, AUPRC =0.957), followed by SVM (AUROC = 0.962, AUPRC =0.949), RF (AUROC = 0.903, AUPRC =0.922) ,KNN(AUROC = 0.883, AUPRC =0.901)In the testing set (Fig. 4).

## Discussion

Although one‐third of the world's population is infected with Mycobacterium tuberculosis, only 5–10% infected people develop active TB disease(ATB). The remaining people still haven’t any symptoms, namely latent tuberculosis infection (LTB) [10]. Discriminative biomarkers that distinguish latent and active tuberculosis can effectively control TB, because early detection, preventive treatment of individuals with LTB and treatment of active TB are the essential proceedings. Previously, Our team found that the monocyte-related indicators in the VCS parameters of tuberculosis patients changed significantly because mononuclear macrophages participate in anti-tuberculosis immunity, and their cell size, internal structure and granules are different in ATB compared to LTB. The three indicators of mean monocyte volume (MV(MO)), mean monocyte volume standard deviation (MV-SD(MO)), and mean monocyte conductivity (MC(MO)) can be combined to obtain superior diagnostic performance (sensitivity: 93.8%, specificity: 93.1%) [5]. Other scholars have found in their research that simultaneous measurement of serum level of interleukine-1β, the mean monocyte volume(MV(MO)) and its distribution width (MV-SD(MO))was able to distinguish active TB infection with an excellent sensitivity of 84.5% and specificity of 90.5% comparable to normal healthy subjects [11].

Nonspecific Immune Responses related by Neutrophil, monocytes, and phagocytes derived from monocytes and Specific Cellular Immunity related by lymphocytes and cytokine would be triggered when the body were invaded by *Mycobacterium tuberculosis*(MTB) [12–14]. The hematology analyzer with VCS (volume, conductivity, light scatter) technology is able to determine the intrinsic biophysical properties of over 8,000 leukocytes in their ‘near native state’ with neither chemical reactions nor fluorescent dye [15]. In this study, we obtained clinically accessible blood routine indicators and the intuitive, and quantifiable parameters related to the inherent detection principle of the instrument with VCS technonogy called VCS parameters. We used the machine learning algorithm of logistic regression(LR), random forest(RF), support vector machine(SVM) and k-nearest neighbor(KNN) to construct prediction models for these 46 features respectively, so as to distinguish active tuberculosis infection(ATB) and latent tuberculosis infection(LTB). The results show that the prediction efficiency of logistic regression and random forest classifier is superior to that of random forest support vector machine and k-nearest neighbor classifier and their AUROC, in turn,is 1,1,0.967 and 0.943. Their AUROC in turn is 0.977, 0.922, 0.949, 0.901 in the testing set. Compared with the biological indicators studied by previous scholars, leukocyte VCS parameters have greater advantages [5, 16, 17]. Furthermore classification effects of machine learning algorithms were better than those elicited by traditional analysis methods [5]. Previously, Some scholars try to increase diagnostic accuracy of tuberculosis with the use of an artificial intelligence approach [18–20], but their research does not involve early diagnosis of tuberculosis. Our further plan and goal is to build an early diagnosis model based on leukocyte VCS parameters by artificial intelligence approach.

This study was subject to the following limitations. In this retrospective study, there were significant differences in age (*p* = *0.002*) and gender (*p* = *0.002*) between the ATB and the LTB. At present, whether age and gender affect the value of the VCS parameters of lymphocytes has not been confirmed and this is indeed necessary for further verification. Secondly, the sample size should be expanded to avoid over-fitting in the classifier training phase.

## Conclusion

In conclusion, the machine learning algorithm classifier based on leukocyte VCS parameters is of great value in identifying active and latent tuberculosis infection. It is very important for accurate and appropriate drug treatment, alleviating diseases and avoiding side effects caused by drug abuse.

## Data Availability

All data and materials are available and potentially shareable on request. The in charge person is Dr. Shaoli Deng. The email is dengshaoli@tmmu.edu.cn.
